# Random effects meta-analysis of COVID-19/S. aureus partnership in co-infection

**DOI:** 10.3205/dgkh000364

**Published:** 2020-11-27

**Authors:** Suleiman Shuaibu Adeiza, Abdulmalik Bello Shuaibu, Gazali Mohammed Shuaibu

**Affiliations:** 1 Department of Pharmaceutical Microbiology, Faculty of Pharmaceutical sciences, Ahmadu Bello University, Zaria, Kaduna, Nigeria; 2 Department of Veterinary Microbiology, Faculty of Veterinary medicine, Usmanu Danfodiyo University, Sokoto, Nigeria; 3 Department of Microbiology, Faculty of sciences, Usmanu Danfodiyo University, Sokoto, Nigeria

**Keywords:** COVID-19, S. aureus, co-infection, meta-analysis, meta-regression

## Abstract

**Aim:** To assess the hypothesis that coinfection with SARS-CoV-2 and *S. aureus* exacerbates morbidity and mortality among patients, the study aims to report the pooled burden of *S. aureus* co-infections in patients hospitalized with COVID-19.

**Methods:** We searched electronic databases and the bibliographies of pertinent papers for articles. We considered studies in which the core result was the number of patients with bacterial (*S. aureus*) co-infection. We performed random effects meta-analysis (REM) because the studies included were sampled from a universe of different populations and high heterogeneity was anticipated. Using the Cochran’s Q statistic, the observed dispersion (heterogeneity) among effect sizes was assessed. The percentage of total variability in the estimates of the effect size was calculated with the I^2^ index. To check for publication bias, the Egger weighted regression, Begg rank correlation and meta-funnel plot were used. We conducted meta-regression analysis to evaluate the variability between our outcomes and the covariates using computational options such as “methods of moments” and then “maximum likelihood” ratio.

**Results:** We included 18 studies and retrieved data for 63,370 patients hospitalized with influenza-like illness, of which about 14,369 (22.67%) tested positive for COVID-19 by rRT-PCR. Of this number, 8,249 (57.4%) patient samples were analyzed. Bacterial, fungal and viral agents were detected in 3,038 (36.8%); *S. aureus* in 1,192 (39.2%). Five studies reported MRSA co-infection. Study quality ranged from 6 to 9 (median 7.1) on a JBI scale. From the meta-analysis, 33.1% patients were found to be coinfected (95%, CI 18.0 to 52.6%, Q=3473: df=17, I^2^=99·48%, p=0.00). The rate of *S. aureus* /COVID-19 co-infection was 25.6% (95% CI: 15.6 to 39.0, Q=783.4, df=17, I^2^=97.702%, p=0.003).The proportion of COVID-19/*S. aureus* co-infected patients with MRSA was 53.9% (95% CI, 24.5 to 80.9, n=66, 5 studies, Q=29.32, df=4, I^2^=86.369%, p=0.000). With the multivariate meta-regression model, study type (p=0.029), quality (p=0.000) and country (p=0.000) were significantly associated with heterogeneity.

**Conclusions:** The pooled rates of *S. aureus* among COVID-19 patients documented in this study support the concern of clinicians about the presence of *S. aureus* in co-infections. Improved antibiotic stewardship can be accomplished through rapid diagnosis by longitudinal sampling of patients.

## Zusammenfassung

**Ziel:** Zur Überprüfung der Hypothese, dass die Koinfektion von SARS-CoV-2 mit *S. aureus* die Morbidität und Mortalität verschlimmert, sollte das Outcome bei Koninfektionen bei mit COVID-19 hospitalisierten Patienten analysiert werden.

**Methode:** Bei der Recherche in elektronischen Datenbanken und Bibliographien wurden alle Studien mit Co-Infektion von COVID-19 und *S. aureus* berücksichtigt. Wir führten eine random effects meta-analysis (REM) durch, da die eingeschlossenen Studien aus verschiedenen Populationen ausgewählt wurden und eine hohe Heterogenität erwartet wurde. Mit Hilfe der Cochran’s Q-Statistik wurde die beobachtete Streuung (Heterogenität) zwischen den Effektgrößen bewertet. Der Prozentsatz der Gesamtvariabilität bei den Schätzungen der Effektgröße wurde mit dem I^2^-Index berechnet. Zur Überprüfung des Publikationsbias wurden die Egger-gewichtete Regression, die Begg-Rang-Korrelation und das Meta-Tunnel-Plot verwendet. Wir führten eine Meta-Regressionsanalyse durch, um die Variabilität zwischen unseren Ergebnissen und den Kovariaten unter Verwendung von Berechnungsoptionen wie „Momentmethoden“ und dann „Maximum-Likelihood“-Verhältnis zu bewerten.

**Ergebnisse:** Es wurden achtzehn Studien mit Daten für 63.370 Patienten eingeschlossen, die mit grippeähnlicher Erkrankung ins Krankenhaus eingewiesen wurden. Davon wurden 14.369 (22,7%) mittels rRT-PCR positiv auf COVID-19 getestet. Hiervon wurden 8.249 (57,4%) Patientenproben analysiert. Bakterielle, pilzliche und virale Erreger wurden bei 3.038 (36,8%), *S. aureus* bei 1.192 (39,2%) nachgewiesen. Fünf Studien berichteten über eine MRSA-Koinfektion. Die Studienqualität reichte von 6 bis 9 (Median 7,1) auf der JBI-Skala. Aus der Meta-Analyse ging hervor, dass 33,1% der Patienten koinfiziert waren (95%, CI 18,0 bis 52,6%, Q=3473: df =17, I^2^=99-48%, p=0,00). Die Rate der Koinfektion von *S. aureus*/COVID-19 betrug 25,6% (95% KI: 15,6 bis 39,0, Q=783,4, df=17, I^2^=97,702%, p=0,003). Der Anteil von COVID-19/*S. aureus* ko-infizierten Patienten mit MRSA betrug 53,9% (95% KI, 24,5 bis 80,9, n=66, 5 Studien, Q=29,32, df=4, I^2^=86,369%, p=0,000). Beim multivariaten Meta-Regressionsmodell waren Studientyp (p=0,029), Qualität (p=0,000) und Land (p=0,000) signifikant mit Heterogenität assoziiert.

**Schlussfolgerung:** Die in dieser Studie dokumentierten gepoolten Raten von *S. aureus* Infektion bei COVID-19-Patienten unterstützen die Besorgnis der Kliniker hinsichtlich des Ausmaßes der Bakterien bei Co-Infektionen. Eine verbesserte Antibiotika-Stewardship kann durch eine schnelle Diagnose mittels Längsschnitt-Stichproben von Patienten erreicht werden.

## Introduction

The morbidity and mortality rate associated with COVID-19 is not unrelated to co-infections with bacterial pathogens [1]. Respiratory and blood culture studies of hospitalized patients with severe acute respiratory coronavirus 2 (SARS-CoV-2) have shown that bacterial infections rather than the direct effects of the virus have resulted in a number of recorded fatalities [2].

*Staphylococcus* *aureus* (*S. aureus*) is persistently and asymptomatically present in the nares of 20% of the human population [3], [4]. In response to physiological changes during infection, bacterial factors involved in the shift of *S. aureus* from commensalism to pathogenesis is poorly understood [5]. The bacteria have been associated with secondary staphylococcal pneumonia following COVID-19 infection [6], [7]. However, the overlap of symptoms makes the identification of co-infected patients and the co-infecting pathogens laborious [8].

Although previous studies investigated the frequency of selected bacterial species in COVID-19 cases [9] and the overall extent of co-infection [10], the pooled rate of *S. aureus* among hospitalized COVID-19 patients is largely undocumented. This study aims to address this issue by conducting a meta-analysis to determine the burden of *S. aureus* co-infections in patients hospitalized with COVID-19. Knowledge about specific etiological agents may reduce the strain on the resources of healthcare systems worldwide and lead to more appropriate treatment and medication, as well as shorter hospitalization.

## Methods

### Search strategy, selection criteria and data extraction

We examined databases for studies that reported data on *S. aureus* and MRSA co-infections in patients with COVID-19 infection. Studies with fewer than 10 participants and case studies were excluded. Searches were performed in Pub Med, Google Scholar, Web of Science and SCOPUS from 1^st^ of January 2020 up to 20^th^ of October 2020. The search terms included: ‘COVID-19 and MRSA’, ‘bacterial infection and MRSA’, ‘*S. aureus* and COVID-19’, ‘SARS-CoV-2 and MRSA or *S. aureus*’ ‘bacterial pathogens and COVID-19’. These were combined with search terms such as ‘hospital’, ‘healthcare’, ‘community-acquired’, ‘hospital-acquired’, ‘bacteremia’, ‘pneumonia’, ‘secondary infections’, ‘supra-infection’, ‘co-infection’. The bibliographies of identifed articles were also searched. The abstracts and full texts of identified studies were screened for eligibility by two reviewers (SSA and ABS). The quality of studies was evaluated using the Joanna Briggs Institute Checklist for Studies Reporting Prevalence Data [11]. Discrepancies in evaluation were settled by consultation with a mediator (SMG). PRISMA (preferred reporting items for systematic reviews and meta-analyses) protocols were used for this analysis [12].

The data gathered from the included studies comprised author’s name, country of study, type of study, setting, culture type, and number of patients with: influenza-like illness (ILI), COVID-19 positive results, co-infections, *S. aureus* co-infections or MRSA co-infections.

### Data analysis and assessment of bias

Random effects meta-analysis (REM) was performed because the studies included were sampled from a universe of different populations and high heterogeneity was anticipated. Comprehensive Meta-Analysis^®^ software, version 3.3070 (Bio-stat, Englewood, NJ, USA), was used to perform the analysis. Using the Cochran’s Q statistic, the observed dispersion (heterogeneity) among effect sizes was assessed. The percentage of total variability in the estimates of the effect size was calculated with the I^2^ index. To check for publication bias, Egger weighted regression and Begg rank correlation methods with a meta-funnel plot were used. A p-value <0.05 was presumed to reflect a statistically significant publication bias. In order to structure the search results and document relevant studies, Zotero desktop^®^ (version 5.0.92) reference software was employed. Sensitivity analyses were carried out to gauge the impact of each study (by omission) on the pooled rates [13].

Meta-regression analysis was conducted to evaluate the variability between our outcomes and the covariates (study type, study quality, setting and country). Covariates were first tested individually in a univariate analysis and then simultaneously in a multiple meta-regression model through the computational options “methods of moments” followed by the “maximum likelihood” ratio. The R^2^ analog was used to compute the total variance of all studies about the grand mean effects. Outlier diagnostics were performed using Cook’s distances, covariance ratios, heterogeneity test statistics and weights (Attachment Fig. S4).

## Results

**Figure 1:**
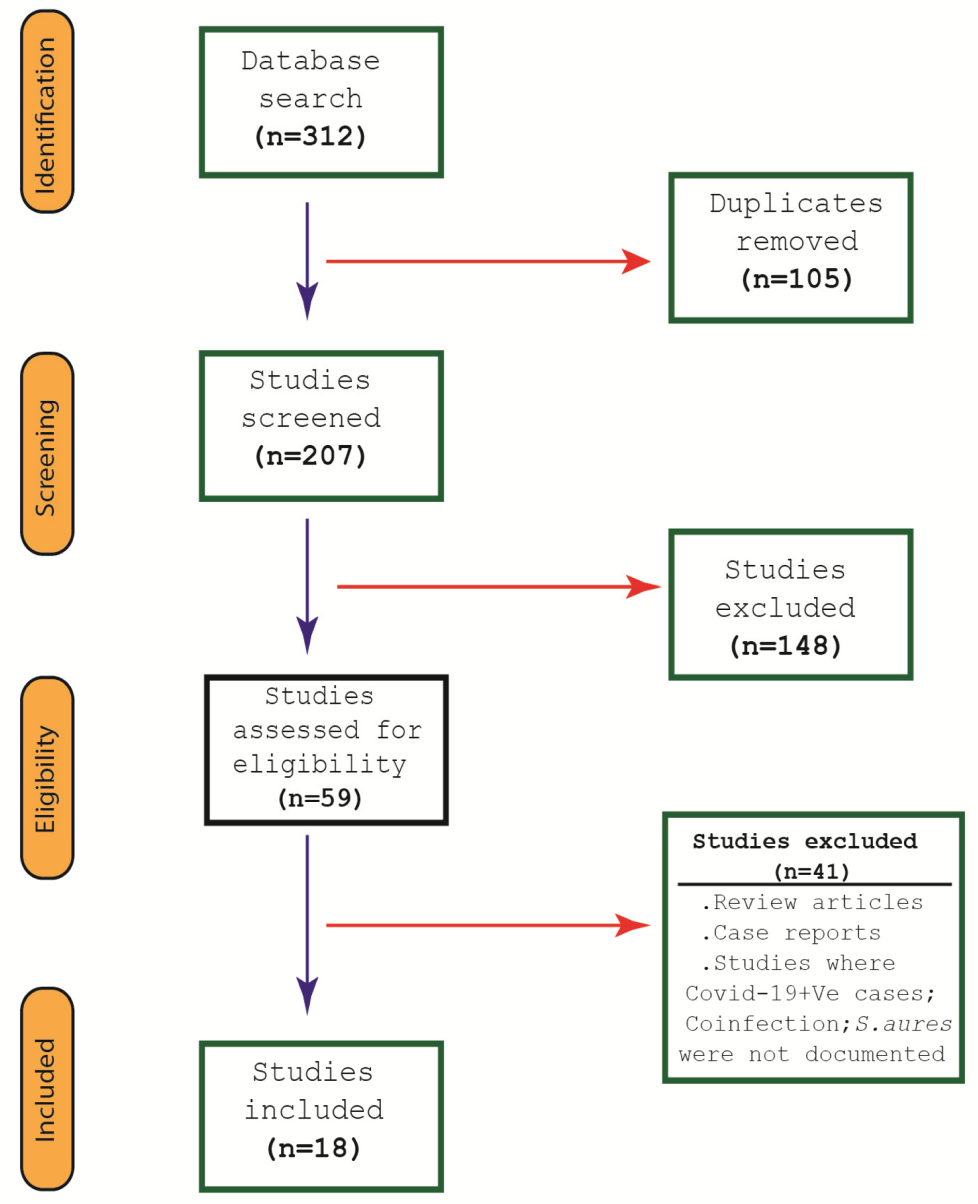
Flow diagram of article selection process

Our search yielded 207 titles after removing duplicates, of which 148 were removed during the initial screening. At this point, the abstracts of the outstanding 59 studies were reviewed, and 41 studies were discarded because they did not meet the inclusion criteria, leaving 18 studies included in the study (Figure 1 (Fig. 1)). The study quality ranged from 6 to 9 (median 7.1) on the Joanna Briggs Institute scale. All included studies reported on *S. aureus*/COVID-19 co-infection amongst hospitalized patients. Table 1 (Tab. 1) provides data on 63,370 patients from the included studies. Five studies were from the United States (27.8%), 2 each from the United Kingdom, China, and Italy (33.3%), 1 each from France, Egypt, Saudi Arabia, Netherlands, Spain, Iran and Russia (38.9%). We retrieved data for the 63,370 patients hospitalized with influenza-like illness, about 14,369 (22.67%) of which tested positive for COVID-19 by rRT-PCR. Of this number, 8,249 (57.4%) of patient samples (respiratory and blood) were analyzed for co-infecting pathogens. Bacterial, fungal and viral agents were detected in 3,038 (36.8%) patients, and *S. aureus* in 1,192 (39.2%) patients. Only five studies reported MRSA co-infection in 66 patients.

**Figure 2:**
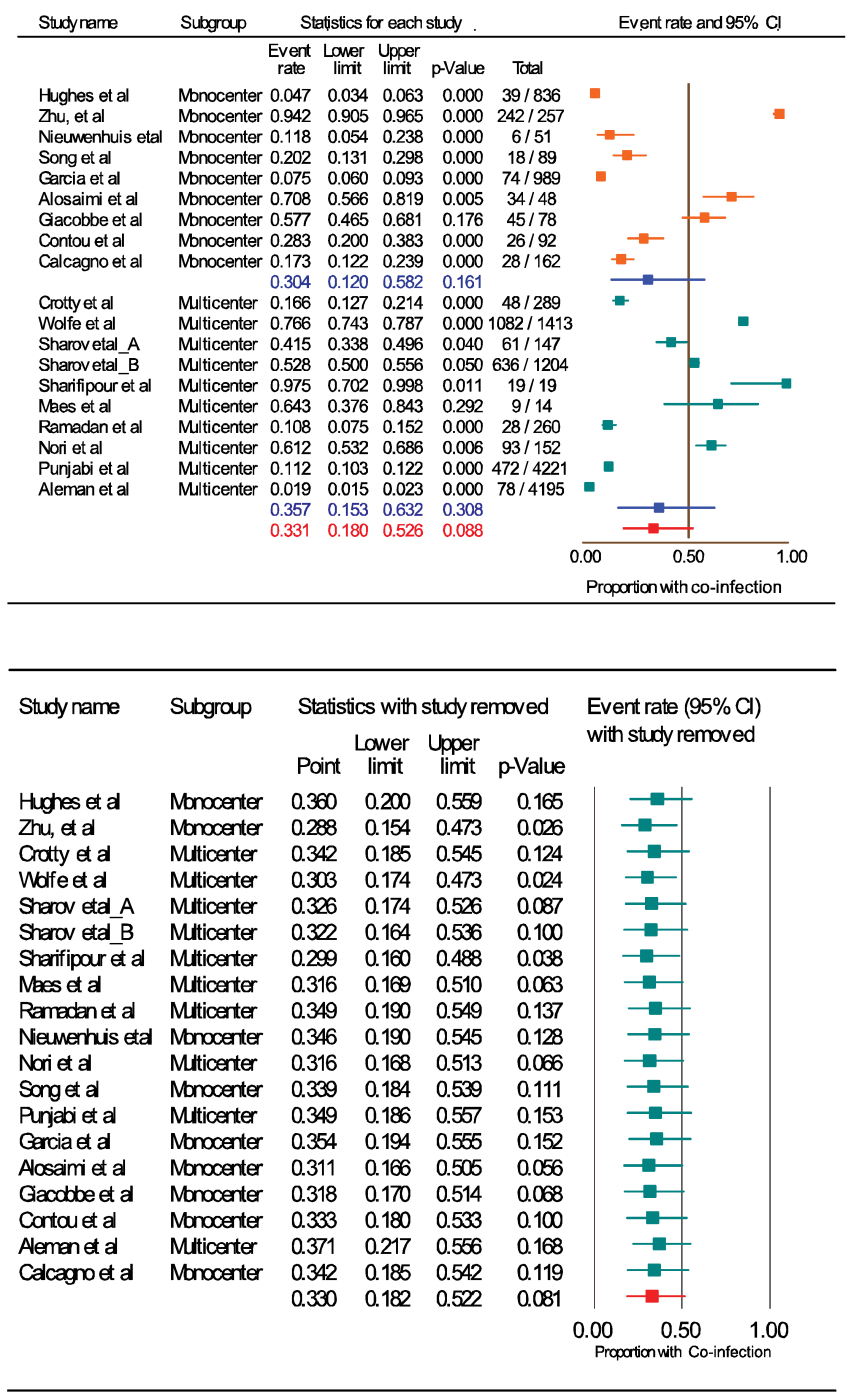
Frequency of co-infection in hospitalized COVID-19 patients (top); sensitivity analysis of the meta-analysis (bottom)

**Figure 3:**
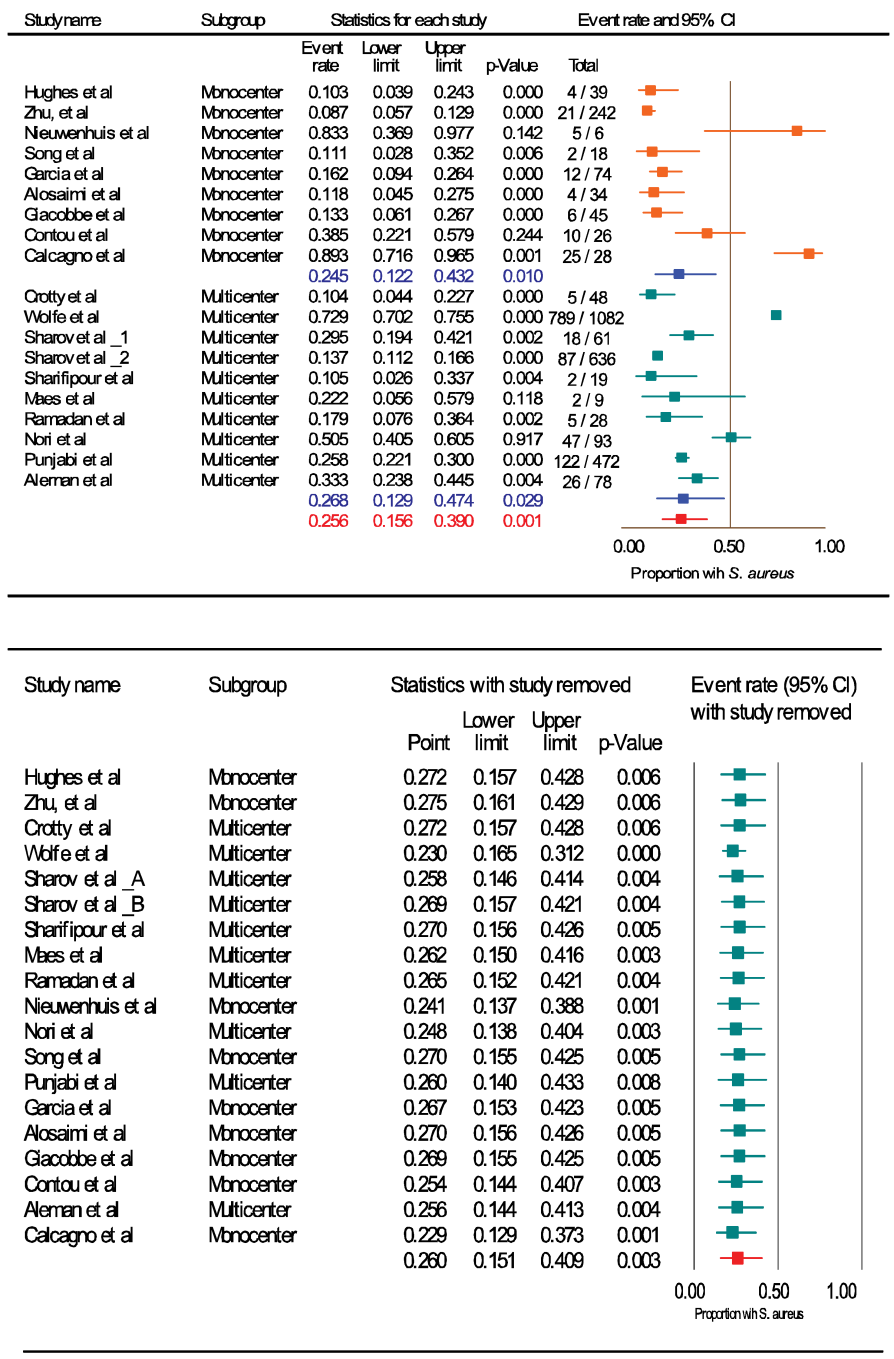
Frequency of *S .aureus* among hospitalized COVID-19 patients with co-infection (Top); sensitivity analysis of the meta-analysis (bottom)

**Figure 4:**
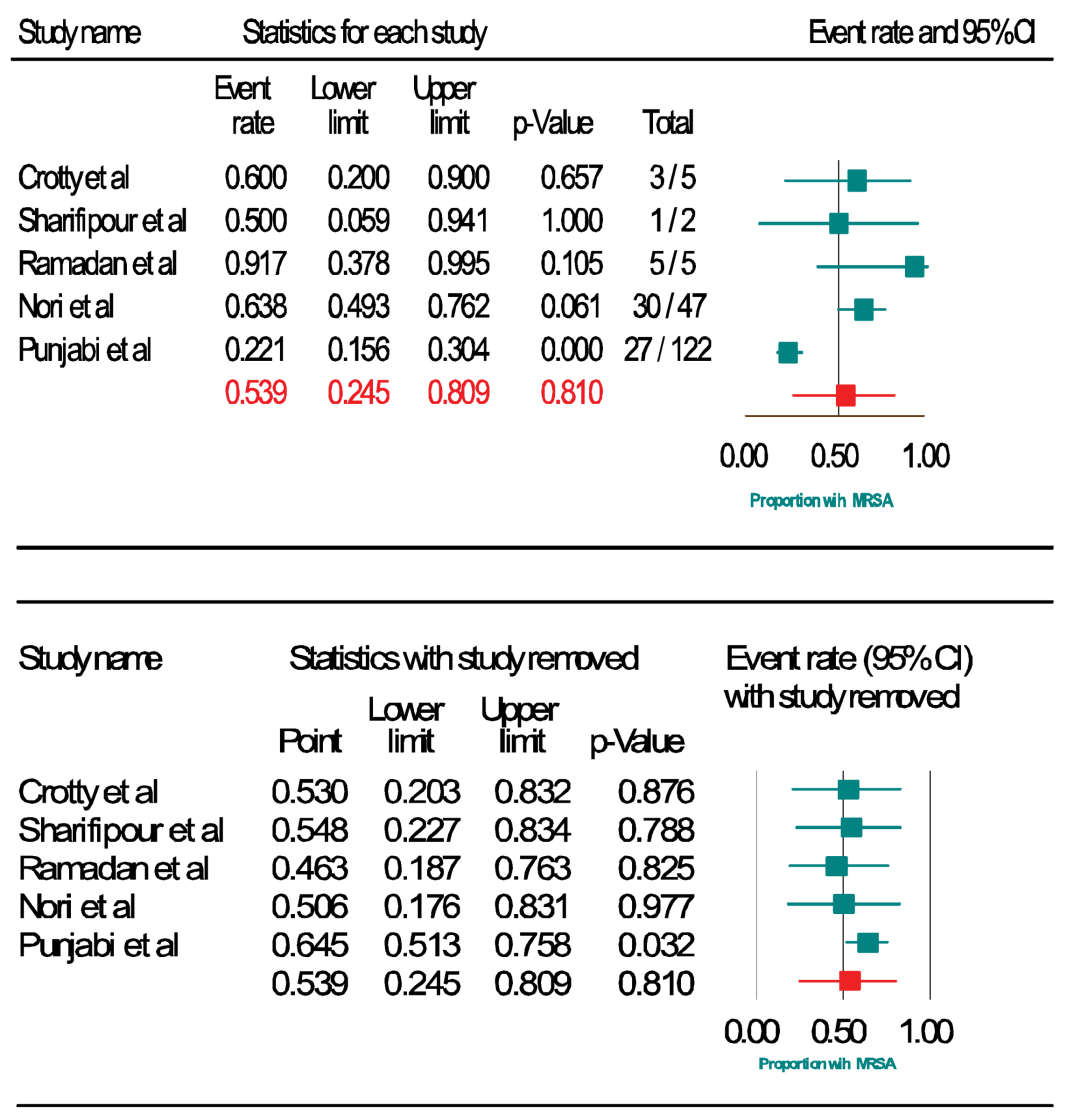
Frequency of MRSA among hospitalized COVID-19 patients with *S. aureus* co-infection (top); sensitivity analysis of the meta-analysis (bottom)

**Figure 5:**
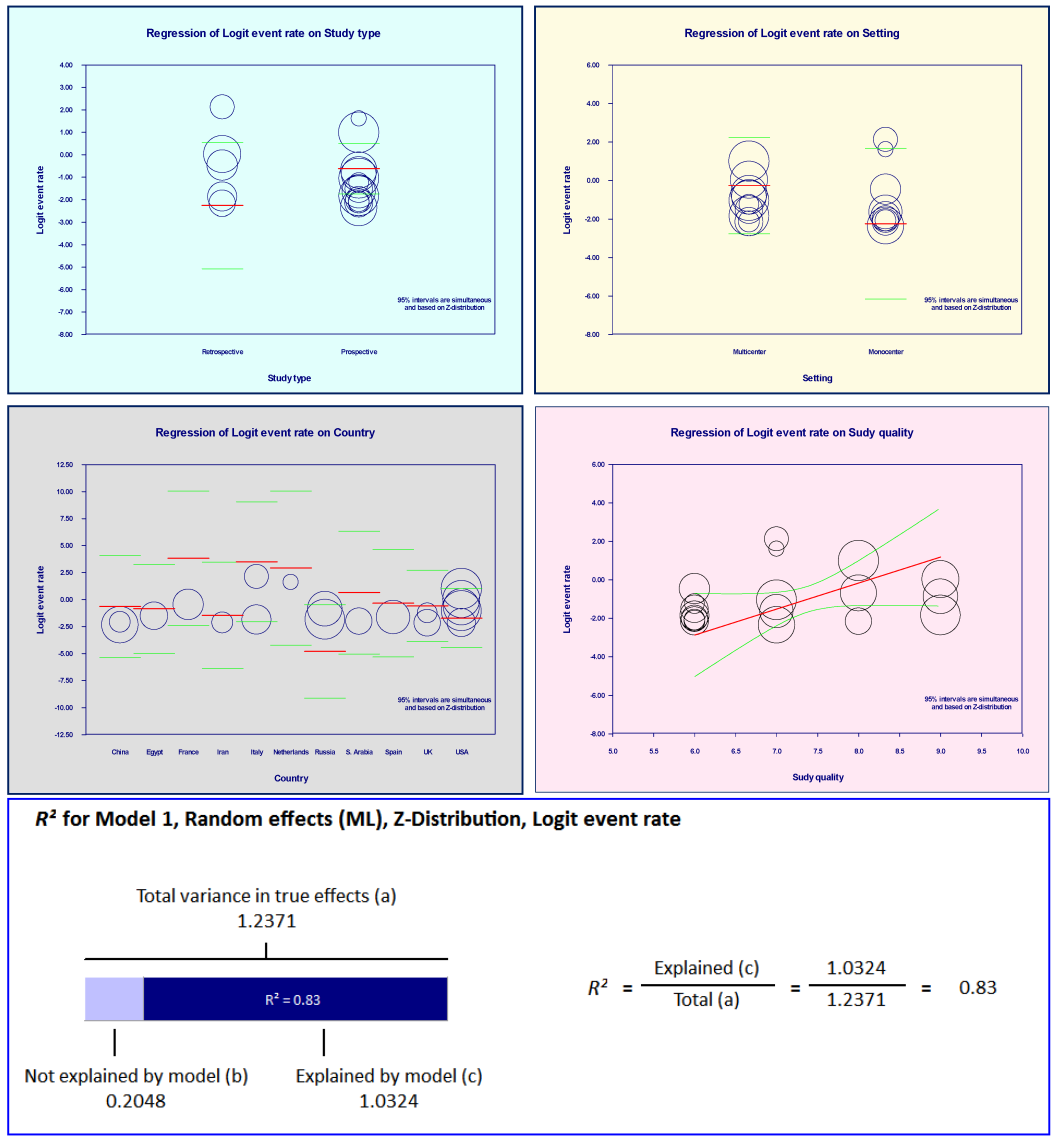
Meta-regression of *S. aureus* co-infection effects and covariates (study type, study quality, study setting, country and combination of covariates)

The Forest plots (Figure 2 (Fig. 2), Figure 3 (Fig. 3), and Figure 4 (Fig. 4)) show the pooled rates of co-infections, *S. aureus*/COVID- 19 co-infection and frequency of MRSA among co-infected patients. Subgroup analysis was performed to evaluate whether the pooled effects differed for mono-center and multicenter studies. The red and blue summary symbols represent the overall and subgroup effect outcome of the analysis, respectively, at a confidence interval of 95%. The squares signify the point estimates of each study and the square's size denotes the weight given in the meta-analysis. From pooled analysis of 18 studies, 33.1% of patients reported co-infection (95%, CI 18.0 to 52.6%, Q=3473: df=17, I^2^=99·48%, p=0.00): mono-center, 30.4% (95% CI, 12.0 to 58.0%); multicenter, 35.7% (95% CI, 15.3 to 63.2%). Additionally, the pooled rate of *S. aureus*/COVID-19 co-infection among patients was 25.6% (95% CI: 15.6 to 39.0, Q=783.4, df=17, I^2^=97.702%, p=0.003): mono-center, 24.5% (95% CI, 12.2 to 43.2%, p=0.010); multicenter, 26.8% (95% CI, 12.9 to 47.4%, p=0.029). The overall pooled proportion of hospitalized COVID-19/*S. aureus* co-infected patients with MRSA was 53.9% (95% CI, 24.5 to 80.9, n=66, 5 studies, Q=29.32, df=4, I^2^=86.369%, p=0.000). The sensitivity analysis did not significantly affect the overall proportion of our results by excluding one study, nor did it affect the heterogeneity. Some indication of publication bias among the analysed studies is shown by the asymmetric distribution of the studies in the funnel plots (Attachement Fig. S1, Fig. S2, and Fig. S3 ), highlighting the statistical heterogeneity observed. Egger’s and Begg’s tests (Attachement Tab. S1 ) did not demonstrate statistical significance for bias in any of the analyses (p>0.05). None of our covariates were statistically significant using the “methods of moment’s” computation method. In the multivariate meta-regression model (maximum likelihood method), study type (p=0.029), study quality (p=0.000) and country of study (p=0.000) were significantly associated with heterogeneity of results (Attachement Fig. S4 ). Of all the moderator variables, only study settings was not statistically significant (0.123). Overall, at a Q-value of 53.79, with df=13 and p=0.000, the covariates were associated with our observed effect. The variance of true effect sizes at any point on the regression line (T^2^) was 0.2048, p≤0.05. Only 83% (R^2^=0.83) of the variance in true effects can be explained by the covariates using our model (Figure 5 (Fig. 5)). 

## Discussion

Normally, an underlying infection is expressed as symptoms. Traditional approaches (qualitative and quantitative) for detecting co-infections are not always effective due to overlapping symptoms. As a result, clinicians prefer empirical antibiotic therapy with an emphasis on etiological staphylococci and streptococcal agents [14], [15]. Although the danger posed by bacterial co-infections in COVID-19 patients is recognized, the extent of co-infection with *S. aureus* has hitherto not been systematically evaluated. This meta-analysis found that overall, in the included studies, more than one-fourth of COVID-19 hospitalized patients had a co-infection (bacterial, fungal or viral), underscoring the need for establishment of protocols for the detection of coinfection to improve clinical data and patient therapy. Similarly, in about one-fourth of recorded co-infections, *S. aureus* was the prevalent co-pathogen. This finding is consistent with a coinfection rate of 25% (*S .aureus*) previously reported by [16]. The finding that MRSA was associated with over half of patients hospitalized with COVID-19/*S. aureus* co-infection is consistent with the prevalence rate of 50% reported by [16] in the 2009 influenza pandemic. The rates observed may be attributable to widespread antibiotic use on skin and nasopharyngeal microbiota, which may degrade the respective ecosystem [17]. The reported MRSA rates may be linked to non-judicious administration of broad-spectrum antibiotics to a large proportion of patients. The sensitivity of *S. aureus* culture methods could have been limited by the excessive use of antibiotics, so that our findings may have underestimated the actual situation.

In our meta-analysis, significant heterogeneity exists, which led us to an enquiry into its origin. The maximum likelihood model explained that covariates such as study type, study quality and country of study were associated with heterogeneity. The unexplained heterogeneity (20.48%) may be due to differences between studies in terms of disease severity, patient co-morbidities, treatment differences, use of antibiotics prior to and during hospitalization, or other unidentifed covariates. 

The strengths of the present study include our use of statistical models to assess the sources of heterogeneity, a systematic search strategy to classify potentially suitable studies from different sources, as well as scrutinizing the supplementary information of preprints and publications up to our search date. While research is ongoing, there are few studies documenting *S. aureus* and MRSA microbiological cultures among COVID-19 positive patients to date. This influenced the distribution of the studies covered. It is also likely that, considering the extraordinary conditions and immense burden on healthcare systems, patients with a suspected secondary infection would not have had extensive microbiological examinations. The data provided by the included studies did not distinguish between the sources of secondary infections and colonizers. This research focused solely on patients who were hospitalized and did not take into account patients who had not been hospitalized.

## Conclusion

The pooled rates of *S. aureus* among COVID-19 patients documented in this study justify the concern of clinicians about the presence of* S. aureus* in co-infections. This data is not sufficient to support widespread- and often inappropriate empirical use of antibiotics in patients hospitalized with COVID-19, as reports of co-infection in admitted patients are scanty. Improved antibiotic stewardship can be accomplished through rapid diagnosis by longitudinal sampling of patients to allow targeted antimicrobial therapy.

## Notes

### Competing interests

The authors declare that they have no competing interests.

### Acknowledgements

We thank Halima Salihu of the Department of Fisheries and Aquaculture, Usmanu Danfodiyo University Sokoto, Nigeria for her supportive remarks.

### Funding

None was received. 

## Supplementary Material

Supplementary material

## Figures and Tables

**Table 1:**
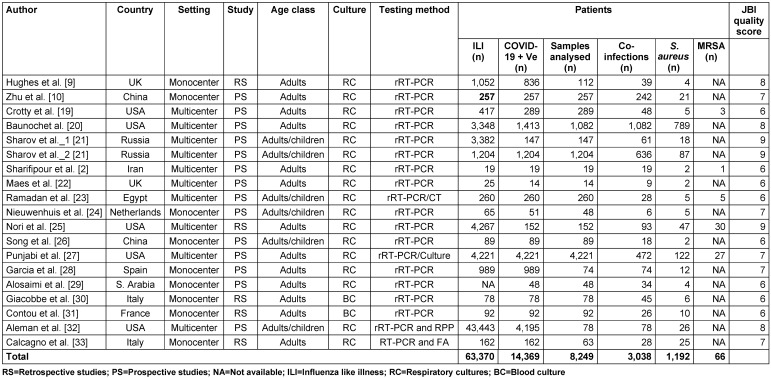
Study characteristics
