# Supplementary material for: Random effects meta-analysis of COVID-19/S. aureus partnership in co-infection
Source: GMS Hyg Infect Control. 2020 Nov 27;15:Doc29. doi: 10.3205/dgkh000364 (PMC7709160; doi:10.3205/dgkh000364)
Supplement: Supplementary material [file HIC-15-29-s-001.pdf]

Supplementary material

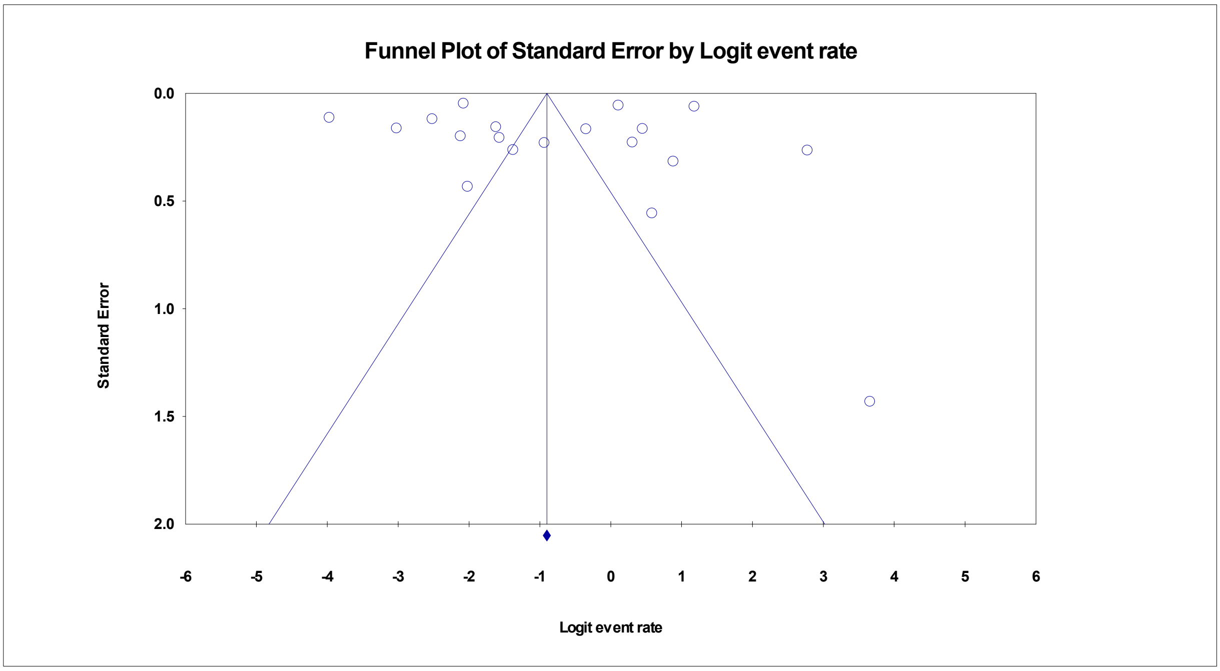

Model		Effect size and 95% interval			Test of null (2-Tail)		Heterogeneity				Tau-squared			
Model	Number Studies	Point estimate	Lower limit	Upper limit	Z-value	P-value	Q-value	df (Q)	P-value	I-squared	Tau Squared	Standard Error	Variance	Tau
Fixed	19	0.289	0.278	0.300	-33.733	0.000	3473.031	18	0.000	99.482	3.002	1.817	3.302	1.733
Random	19	0.330	0.182	0.522	-1.743	0.081								

Figure S1: Funnel plot of standard error (y-axis) against rate of co-infection in hospitalized COVID-19 patients

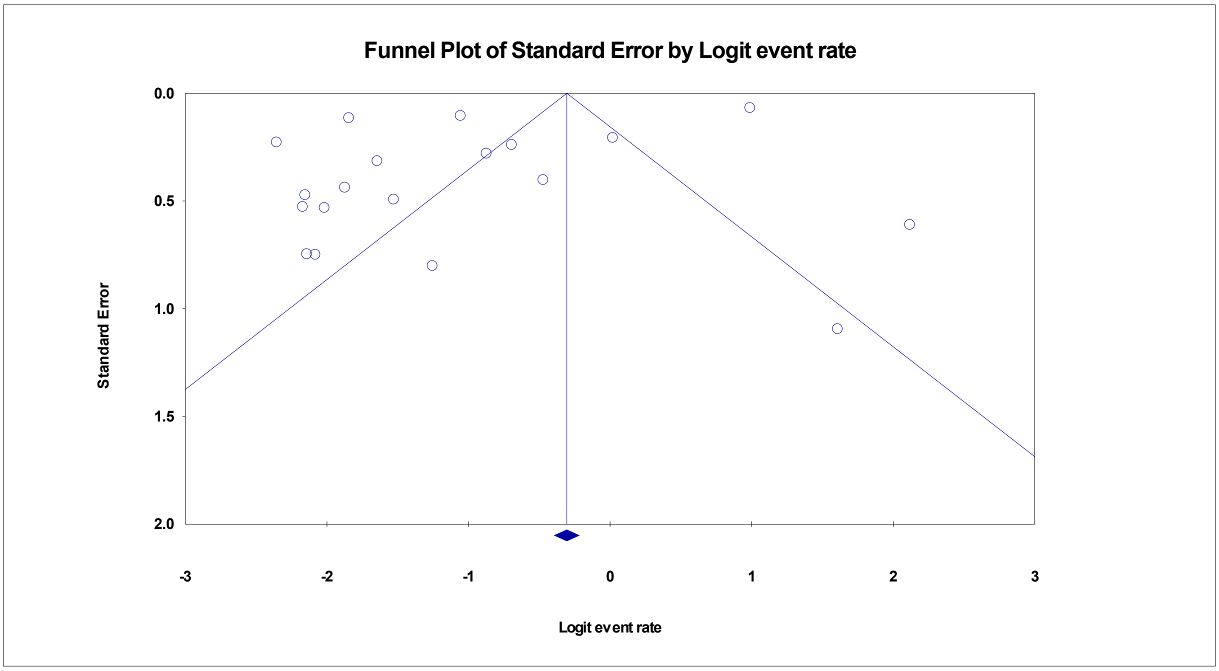

Model		Effect size and 95% interval			Test of null (2-Tail)		Heterogeneity				Tau-squared			
Model	Number Studies	Point estimate	Lower limit	Upper limit	Z-value	P-value	Q-value	df (Q)	P-value	I-squared	Tau Squared	Standard Error	Variance	Tau
Fixed	19	0.424	0.403	0.446	-6.822	0.000	783.405	18	0.000	97.702	2.043	1.391	1.936	1.429
Random	19	0.260	0.151	0.409	-3.022	0.003								

Figure S2: Funnel plot of standard error (y-axis) against rate of *S. aureus* co-infection in hospitalized COVID-19 patients.

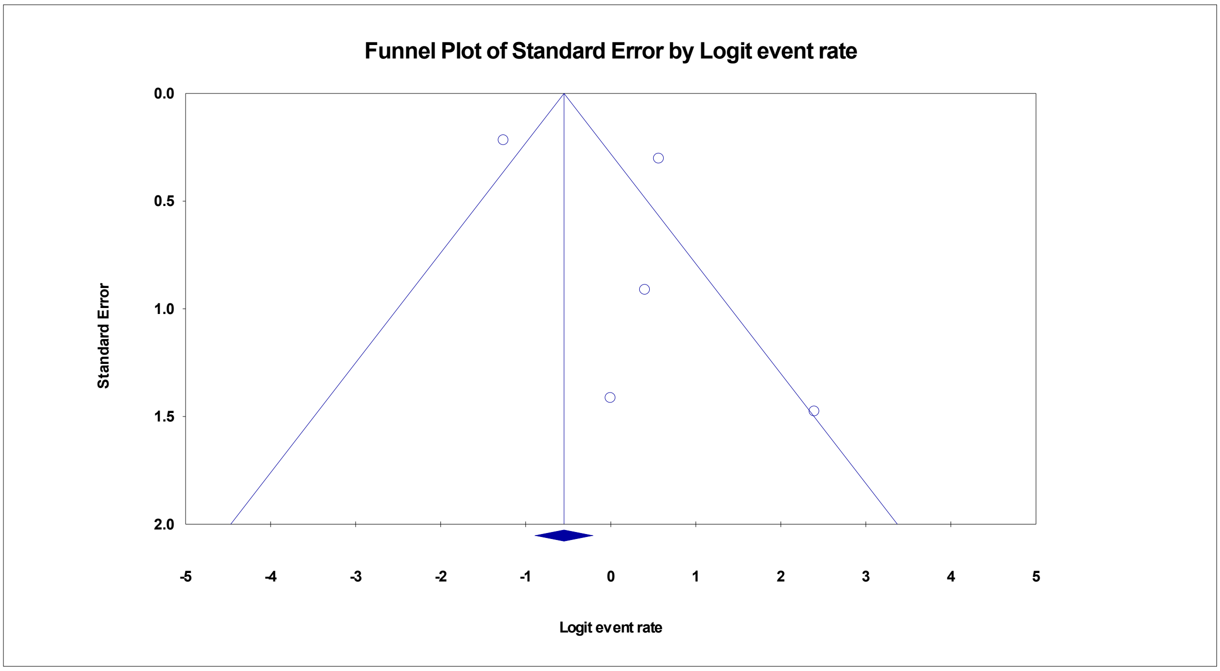

Model		Effect size and 95% interval			Test of null (2-Tail)		Heterogeneity				Tau-squared			
Model	Number Studies	Point estimate	Lower limit	Upper limit	Z-value	P-value	Q-value	df (Q)	P-value	I-squared	Tau Squared	Standard Error	Variance	Tau
Fixed	5	0.366	0.292	0.447	-3.206	0.001	29.332	4	0.000	86.363	1.445	1.802	3.246	1.202
Random	5	0.539	0.245	0.809	0.240	0.810								

Figure S3: Funnel plot of standard error (y-axis) against rate of MRSA in hospitalized COVID-19 patients with *S. aureus* co-infection.

Attachment 1 to: Adeiza SS, Shuaibu AB, Shuaibu GM. Random effects meta-analysis of Covid-19/S. aureus partnership in co-infection. GMS Hyg Infect Control. 2020;15:Doc29. DOI: 10.3205/dgkh000364

Table S1: Results of Begg's rank correlation test and Egger's weighted regression analysis.

	**Coinfection**	***S. aureus***	**MRSA**
**Begg and Mazumdar rank correlation**			
**Kendall's S statistic (P-Q)**	39.00000	41.00000	2.00000
**Kendall's tau without continuity correction**			
Tau	0.22807	0.23977	0.20000
z-value for tau	1.36444	1.43441	0.48990
P-value (1-tailed)	0.08621	0.07573	0.31210
P-value (2-tailed)	0.17243	0.15146	0.62421
**Kendall's tau with continuity correction**			
Tau	0.22222	0.23392	0.10000
z-value for tau	1.32945	1.39942	0.24495
P-value (1-tailed)	0.09185	0.08084	0.40325
P-value (2-tailed)	0.18370	0.16169	0.80650
**Egger's regression intercept**			
Intercept	-0.33605	-3.98117	2.09094
Standard error	5.36750	2.10865	1.95861
95% lower limit (2-tailed)	-11.66048	-8.43002	-4.14225
95% upper limit (2-tailed)	10.98838	0.46769	8.32413
t-value	0.06261	1.88802	1.06756
df	17.00000	17.00000	3.00000
P-value (1-tailed)	0.47540	0.03811	0.18200
P-value (2-tailed)	0.95081	0.07621	0.36400

Main results for Model 1, Random effects (ML), Z-Distribution, Logit event rate

	**Intercept**	-11.0668	2.9724	-16.8926	-5.2411	-3.72	0.0002	
	**Setting: Monocenter**	-1.9840	1.2878	-4.5081	0.5401	-1.54	0.1234	
	**Study type**	1.6374	0.7466	0.1741	3.1007	2.19	0.0283	
**Country**	**Country: Egypt**	-0.2119	1.5150	-3.1814	2.7575	-0.14	0.8888	Q=40.39, df=10, p=0.0000
	**Country: France**	4.4655	1.1836	2.1456	6.7854	3.77	0.0002	
	**Country: Iran**	-0.8259	1.6158	-3.9928	2.3409	-0.51	0.6092	
	**Country: Italy**	4.1385	1.0495	2.0815	6.1955	3.94	0.0001	
	**Country: Netherlands**	3.5550	1.2662	1.0733	6.0367	2.81	0.0050	
	**Country: Russia**	-4.1634	1.6231	-7.3447	-0.9822	-2.57	0.0103	
	**Country: S. Arabia**	1.2832	0.8570	-0.3965	2.9629	1.50	0.1343	
	**Country: Spain**	0.3033	0.7090	-1.0864	1.6930	0.43	0.6688	
	**Country: UK**	0.0614	0.9997	-1.8981	2.0208	0.06	0.9511	
	**Country: USA**	-1.0846	1.4612	-3.9485	1.7793	-0.74	0.4579	
	**Sudy quality**	1.3526	0.3099	0.7452	1.9599	4.36	0.0000	

Statistics for Model 1

Test of the model: Simultaneous test that all coefficients (excluding intercept) are zero

Q = 53.79, df = 13, p = 0.0000

Goodness of fit: Test that unexplained variance is zero

Tau^2 = 0.2048, Tau = 0.4525, I^2 = 93.21%, Q = 73.63, df = 5, p = 0.0000

Comparison of Model 1 with the null model

Total between-study variance (intercept only)

Tau^2 = 1.2371, Tau = 1.1123, I^2 = 97.70%, Q = 783.41, df = 18, p = 0.0000

Proportion of total between-study variance explained by Model 1

R^2 analog = 0.83

Number of studies in the analysis 19

Figure S4: Main random effects meta-regression (maximum likelihood) results for four covariates pooled together

Study	Observed	Predicted	Residual	Leverage	Student Residual	Jacknifed Residual	Cook's Distance	DF Fits	Variance	Tau^2	Sum	Weight	Pct Wt	Pct Wt
Hughes et al | Monocenter	-2.1691	-2.1691	0.0000	1.0000	0.0000	0.0000	0.0000	0.0000	0.2786	0.2048	0.4833	2.0689	0.0393	
Zhu, et al | Monocenter	-2.3536	-1.9456	-0.4081	0.7727	-1.6885	-2.3038	0.6922	-4.2473	0.0521	0.2048	0.2569	3.8924	0.0739	
Crotty et al | Multicenter	-2.1518	-2.3988	0.2470	0.5910	0.5903	0.5474	0.0360	0.6581	0.2233	0.2048	0.4280	2.3363	0.0443	
Wolfe et al | Multicenter	0.9906	0.3064	0.6842	0.4711	2.0558	4.6741	0.2689	4.4112	0.0047	0.2048	0.2094	4.7744	0.0906	
Sharov et al _A | Multicenter	-0.8708	-1.4199	0.5491	0.4347	1.3714	1.5530	0.1033	1.3619	0.0788	0.2048	0.2836	3.5263	0.0669	
Sharov et al _B | Multicenter	-1.8422	-1.4199	-0.4223	0.5653	-1.3714	-1.5530	0.1747	-1.7710	0.0133	0.2048	0.2181	4.5854	0.0870	
Sharifipour et al | Multicenter	-2.1401	-2.1401	0.0000	1.0000	0.0000	0.0000	0.0000	0.0000	0.5588	0.2048	0.7636	1.3096	0.0248	
Maes et al | Multicenter	-1.2528	-1.2528	-0.0000	1.0000	0.0000	0.0000	0.0000	0.0000	0.6429	0.2048	0.8476	1.1798	0.0224	
Ramadan et al | Multicenter	-1.5261	-1.5261	0.0000	1.0000	0.0000	0.0000	0.0000	0.0000	0.2435	0.2048	0.4482	2.2309	0.0423	
Nieuwenhuis et al | Monocenter	1.6094	1.6094	-0.0000	1.0000	0.0000	0.0000	0.0000	0.0000	1.2000	0.2048	1.4048	0.7119	0.0135	
Nori et al | Multicenter	0.0215	0.0215	-0.0000	1.0000	0.0000	0.0000	0.0000	0.0000	0.0430	0.2048	0.2478	4.0358	0.0766	
Song et al | Monocenter	-2.0794	-3.2981	1.2187	0.3211	1.6885	2.3038	0.0963	1.5844	0.5625	0.2048	0.7673	1.3033	0.0247	
Punjabi et al | Multicenter	-1.0539	-1.0462	-0.0077	0.3697	-0.0209	-0.0187	0.0000	-0.0143	0.0111	0.2048	0.2158	4.6334	0.0879	
Garcia et al | Monocenter	-1.6422	-1.6422	-0.0000	1.0000	0.0000	0.0000	0.0000	0.0000	0.0995	0.2048	0.3042	3.2870	0.0624	
Alosaimi et al | Monocenter	-2.0149	-2.0149	-0.0000	1.0000	0.0000	0.0000	0.0000	0.0000	0.2833	0.2048	0.4881	2.0487	0.0389	
Giacobbe et al | Monocenter	-1.8718	-0.7970	-1.0748	0.6329	-2.8151	-1.#INF	0.9760	-1.#INF	0.1923	0.2048	0.3971	2.5184	0.0478	
Contou et al | Monocenter	-0.4700	-0.4700	0.0000	1.0000	0.0000	0.0000	0.0000	0.0000	0.1625	0.2048	0.3673	2.7228	0.0517	
Aleman et al | Multicenter	-0.6931	0.3064	-0.9995	0.3759	-2.4696	-1.#INF	0.2624	-1.#INF	0.0577	0.2048	0.2625	3.8101	0.0723	
Calcagno et al | Monocenter	2.1203	0.5555	1.5647	0.4656	2.8151	1.#INF	0.4931	1.#INF	0.3733	0.2048	0.5781	1.7298	0.0328	

Figure S4: Diagnosis of random effects model
